# Association Between Patient Body Mass Index and Preferred Interpersonal Distance From Pharmacists

**DOI:** 10.7759/cureus.116873

**Published:** 2026-09-24

**Authors:** Haruno Yamagiwa, Saori Gocho, Yukina Miyagi, Yuka Miyachi, Chika Nakayama, Taeyuki Oshima

**Affiliations:** 1 Graduate School of Pharmaceutical Science, Kinjo Gakuin University, Nagoya, JPN; 2 Department of Pharmacy, Nagoya Ekisaikai Hospital, Nagoya, JPN; 3 College of Pharmacy, Kinjo Gakuin University, Nagoya, JPN; 4 Department of Pharmacy, Gifu University of Medical Science, Kani, JPN

**Keywords:** body mass index: bmi, cancer outpatient, interpersonal distance, medication instruction, pharmacist

## Abstract

Appropriate interpersonal distance is an important element of nonverbal communication during medication counseling by pharmacists. Although interpersonal distance can be influenced by several factors such as sex, age, and psychological condition, the relationship between patients’ body mass index (BMI) and their preferred interpersonal distance from pharmacists remains unclear.

This study aimed to examine the association between patients' BMI and their preferred interpersonal distance from pharmacists. A total of 95 outpatients receiving anticancer drug therapy at the Nagoya Ekisaikai Hospital participated in this study. The preferred interpersonal distance was measured using both approach and withdrawal methods. The participants were divided into two groups according to their BMI: < 25 kg/m² and ≥ 25 kg/m². The preferred interpersonal distances of the two groups were then compared. Additional analyses were conducted to examine the combined effects of BMI, sex, and age.

No significant difference in the preferred interpersonal distance was found between the two BMI groups. The combined analysis of sex and BMI also showed no statistically significant differences, although a possible trend was observed among the groups (p = 0.06). Similarly, no significant differences were identified in the combined analysis of age and BMI.

No statistically significant association between BMI and preferred interpersonal distance was identified in this sample. However, the possible relationship between sex and BMI should be examined further in studies with larger sample sizes.

## Introduction

Pharmacists play an important role in supporting appropriate pharmacotherapy through patient communication. Effective pharmacist-patient communication helps pharmacists provide medication information, assess patients' understanding and adherence, and identify treatment-related concerns [1,2]. In Japan, pharmacists are increasingly expected to provide patient-centered care to community-based healthcare professionals [3]. Therefore, establishing a comfortable communication environment for pharmaceutical care is important.

Communication involves both verbal and non-verbal elements. Non-verbal communication includes facial expressions, body movements, posture, eye contact, and interpersonal distance [4,5]. Among these factors, interpersonal distance is an important component of communication because an inappropriate distance may cause discomfort or anxiety and affect interpersonal interactions.

The concept of interpersonal distance has been studied as a part of personal space. Hall classified the interpersonal distance into four zones: intimate, personal, social, and public distances [6]. Sommer also described personal space as an invisible area surrounding an individual, and suggested that intrusion into this space may cause psychological discomfort [7]. Previous studies have shown that the preferred interpersonal distance may be influenced by various factors, including sex, age, personality, familiarity, and characteristics of the approaching person [8-10]. Psychological and physiological responses may also occur when another person enters an individual's personal space [11].

Interpersonal distance is also influenced by another person's impressions and emotional expressions. Iachini et al. reported that interpersonal distance during virtual interactions varies according to a person’s moral impressions [12]. Ruggiero et al. demonstrated that facial expressions affected both peri-personal and interpersonal spaces [13]. These findings suggest that interpersonal distance is not fixed but changes according to the characteristics of individuals and social situations.

The importance of personal space has also been examined in healthcare settings. Studies involving patients with schizophrenia and hospitalized patients have suggested that appropriate interpersonal distance may differ according to patient characteristics and clinical situations [14,15]. Furthermore, the physical environment and spatial limitations may affect the perception of interpersonal distance [16,17]. 

However, the physical characteristics of these patients have received limited attention. In particular, whether body size, including body mass index (BMI), is associated with preferred interpersonal distance from pharmacists remains unclear. Recent evidence also indicates that interpersonal distance changes across the lifespan, emphasizing the importance of considering individual characteristics when evaluating personal space [18].

Therefore, this study aimed to investigate whether patient BMI influences preferred interpersonal distance from pharmacists during medication counseling. We also examined whether other patient characteristics were associated with preferred distance. Clarifying these relationships may help pharmacists better understand individual differences in personal space and provide more comfortable communication for each patient.

This article was previously presented as a meeting abstract at the 35th Annual Meeting of the Japanese Society of Pharmaceutical Health Care and Sciences on November 23, 2025.

## Materials and methods

Participants

This study included 95 outpatients who received anticancer therapy at Nagoya Ekisaikai Hospital. Potential participants were directly approached by the investigators and invited to participate in the study. Of these, 52 were male, and 43 were female. The median age was 71 years, and the median BMI was 22.8 kg/m². Measurements were conducted at the bedside in the chemotherapy center during periods that did not interfere with routine clinical care. Patients who were deaf or unable to understand the purpose of the study were excluded. The study period was from February 2020 to December 2021, with an interruption during the study period owing to the COVID-19 pandemic. Informed consent was obtained from all participants before participation.

Ethical considerations

This study was approved by the Ethics Committee of Nagoya Ekisaikai Hospital (Approval No. 2019-055). The purpose and procedures of the study were explained to all participants, and informed consent was obtained before participation.

Measurement of interpersonal distance

Measurements were performed by three pharmacists: one female pharmacist (45 years old, height 161 cm, weight 48 kg, BMI 18.52 kg/m²) and two male pharmacists (both 36 years old; height 174 cm, weight 65 kg, BMI 21.47 kg/m²; and height 172 cm, weight 72 kg, BMI 24.34 kg/m²). Of the 95 patients, 41 were assessed by a male pharmacist and 54 by the female pharmacist. All three pharmacists had a BMI < 25 kg/m², and the measurement procedure and conditions were standardized across patient assessments. Before patient measurements, the three pharmacists practiced the measurement procedure together to standardize the technique.

Measurements were conducted at the bedside in the chemotherapy center during periods that did not interfere with routine clinical care. The patients were placed in the supine position, and the position of the pharmacist relative to the patient was standardized at an angle of 45°. The pharmacist's eye level was adjusted to the patient's eye level to reduce psychological pressure during communication.

Interpersonal distance was measured using both the approach and withdrawal methods. In the approach method, the pharmacist started from a position sufficiently far from the patient and moved closer in half-step increments. Patients were instructed to verbally indicate when the distance felt “just right” for receiving medication counseling, and the pharmacist stopped at that point. The distance between the tip of the pharmacist's chin and the tip of the patient's chin was then measured using a laser distance meter (Zamo II, Bosch).

In the withdrawal method, the pharmacist started from a close position and moved away from the patient in half-step increments. Patients were similarly instructed to verbally indicate when the distance felt “just right” for receiving medication counseling, and the distance was measured at the point of this verbal response using the same method.

The mean value of the distances obtained using the approach and withdrawal methods was defined as the optimal interpersonal distance for each patient.

Statistical analysis

Patients were divided into two groups according to their BMI: < 25 kg/m² and ≥ 25 kg/m². The cutoff value of 25 kg/m² was selected based on the criterion for obesity established by the Japan Society for the Study of Obesity [19]. The optimal interpersonal distance was compared between the two groups using the Mann-Whitney U test.

Patients were also divided into four groups according to combinations of BMI and sex and combinations of BMI and age (< 75 years or ≥ 75 years). Differences among the four groups were examined using the Kruskal-Wallis test. In addition to the categorical analysis, the association between BMI as a continuous variable and optimal interpersonal distance was examined using Spearman's rank correlation coefficient. Statistical significance was set at p < 0.05.

## Results

A total of 95 patients were included in the analysis. The median age was 71 years, and the median BMI was 22.8 kg/m². Patient characteristics, including sex and BMI categories, are summarized in Table 1.

**Table 1:** Baseline Characteristics of the Study Participants BMI: body mass index.

Characteristic	Total (N = 95)
Sex, n (%)	
Male	52 (54.7)
Female	43 (45.3)
Age, median, years	71
BMI, median, kg/m²	22.8
BMI < 25 kg/m², n (%)	70 (73.7)
BMI ≥ 25 kg/m², n (%)	25 (26.3)

Comparison of interpersonal distance according to BMI

Figure 1 shows the optimal interpersonal distance according to patient BMI. Descriptive statistics for optimal interpersonal distance are presented in Table 2. The median optimal interpersonal distance was 98.3 cm [interquartile range (IQR), 81.6-109.4 cm] in the BMI < 25 kg/m² group and 97.7 cm (IQR, 89.3-111.0 cm) in the BMI ≥ 25 kg/m² group. No statistically significant difference was found between the two groups (Mann-Whitney U test, U = 913.000, p = 0.748). When BMI was analyzed as a continuous variable, no statistically significant correlation was observed between BMI and optimal interpersonal distance (Spearman's ρ = −0.031, p = 0.764, n = 95).

**Figure 1:**
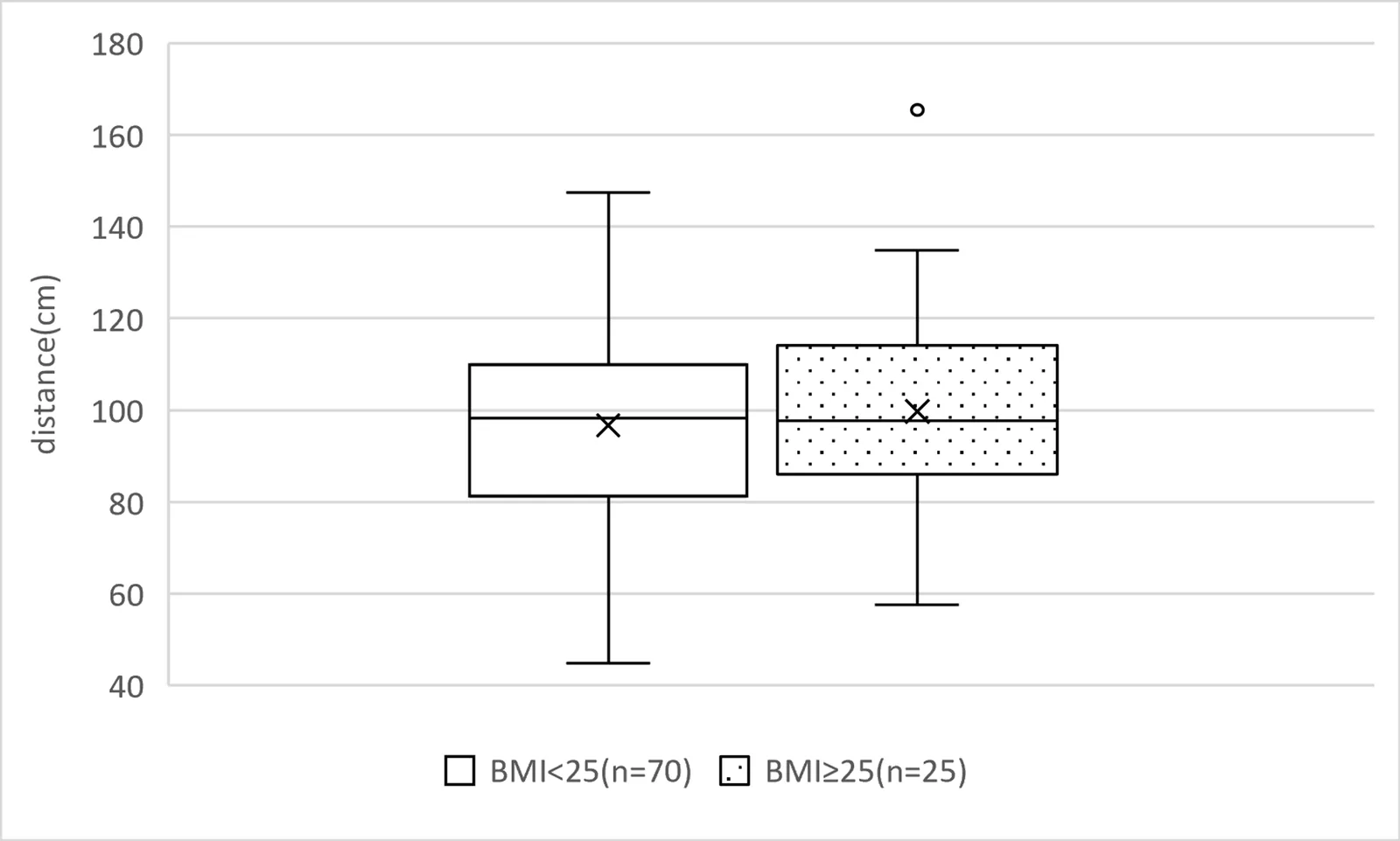
Comparison of optimal interpersonal distance from the pharmacist according to patient BMI The box plots show the distribution of preferred interpersonal distance in patients with BMI < 25 kg/m² and BMI ≥ 25 kg/m². The horizontal line within each box represents the median, and the box represents the interquartile range. The cross indicates the mean, and circles indicate outliers. BMI: body mass index.

**Table 2:** Optimal Interpersonal Distance According to BMI, Sex, and Age BMI: body mass index; IQR: interquartile range. Optimal interpersonal distance was defined as the mean of the distances obtained using the approach and withdrawal methods.

Group	n	Median (cm)	IQR (cm)
BMI < 25 kg/m²	70	98.3	81.6–109.4
BMI ≥ 25 kg/m²	25	97.7	89.3–111.0
Male, BMI < 25 kg/m²	37	101.2	88.5–113.0
Male, BMI ≥ 25 kg/m²	15	101	94.0–118.9
Female, BMI < 25 kg/m²	33	87.8	76.2–105.2
Female, BMI ≥ 25 kg/m²	10	90.1	79.4–97.9
Age < 75 years, BMI < 25 kg/m²	45	98.6	82.7–109.1
Age < 75 years, BMI ≥ 25 kg/m²	19	99.5	91.6–118.9
Age ≥ 75 years, BMI < 25 kg/m²	25	98	79.9–112.8
Age ≥ 75 years, BMI ≥ 25 kg/m²	6	83.3	68.6–92.0

These results indicate that no statistically significant association between BMI and preferred interpersonal distance was observed in this sample.

Analysis according to sex and BMI

Figure 2 shows the comparison of optimal interpersonal distance among the four groups based on sex and BMI. The median optimal interpersonal distances were 101.2 cm (IQR, 88.5-113.0 cm) in male patients with BMI < 25 kg/m², 101.0 cm (IQR, 94.0-118.9 cm) in male patients with BMI ≥ 25 kg/m², 87.8 cm (IQR, 76.2-105.2 cm) in female patients with BMI < 25 kg/m², and 90.1 cm (IQR, 79.4-97.9 cm) in female patients with BMI ≥ 25 kg/m² (Table 2). No statistically significant differences were found among the four groups using the Kruskal-Wallis test [χ²(3) = 7.399, p = 0.06]. However, a possible trend toward a difference among the groups was observed.

**Figure 2:**
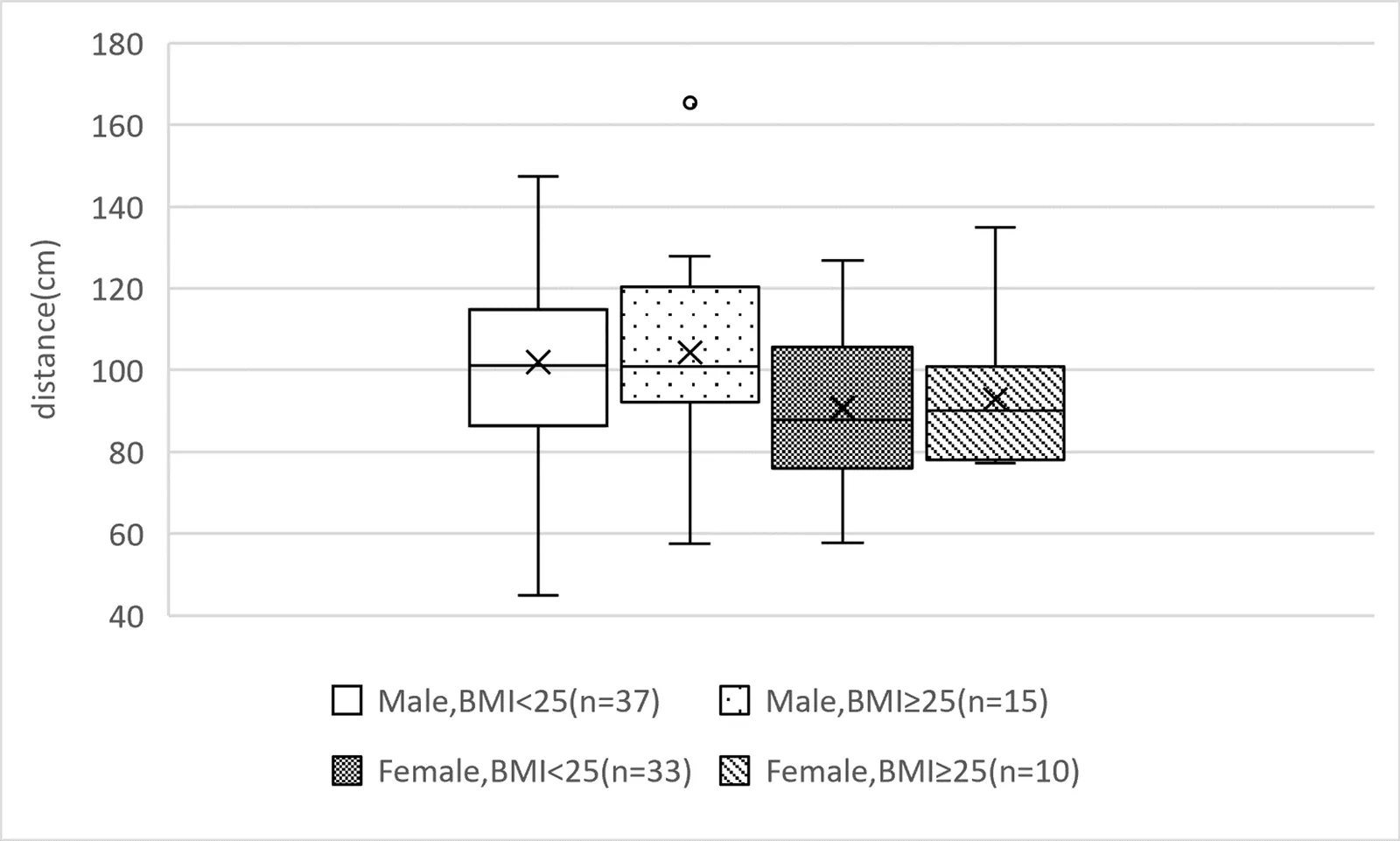
Comparison of optimal interpersonal distance from the pharmacist according to patient sex and BMI The box plots show the distribution of preferred interpersonal distance in four groups according to patient sex and BMI: male/BMI < 25 kg/m², male/BMI ≥ 25 kg/m², female/BMI < 25 kg/m², and female/BMI ≥ 25 kg/m². The horizontal line within each box represents the median, and the box represents the interquartile range. The cross indicates the mean, and circles indicate outliers. The four groups were compared using the Kruskal-Wallis test [χ²(3) = 7.399, p = 0.06]. BMI: body mass index.

Analysis according to age and BMI

Figure 3 shows the comparison of optimal interpersonal distance among the four groups based on age and BMI. The median optimal interpersonal distances were 98.6 cm (IQR, 82.7-109.1 cm) in patients aged < 75 years with BMI < 25 kg/m², 99.5 cm (IQR, 91.6-118.9 cm) in patients aged < 75 years with BMI ≥ 25 kg/m², 98.0 cm (IQR, 79.9-112.8 cm) in patients aged ≥ 75 years with BMI < 25 kg/m², and 83.3 cm (IQR, 68.6-92.0 cm) in patients aged ≥ 75 years with BMI ≥ 25 kg/m² (Table 2). No statistically significant differences were found among the four groups using the Kruskal-Wallis test [χ²(3) = 5.785, p = 0.123].

**Figure 3:**
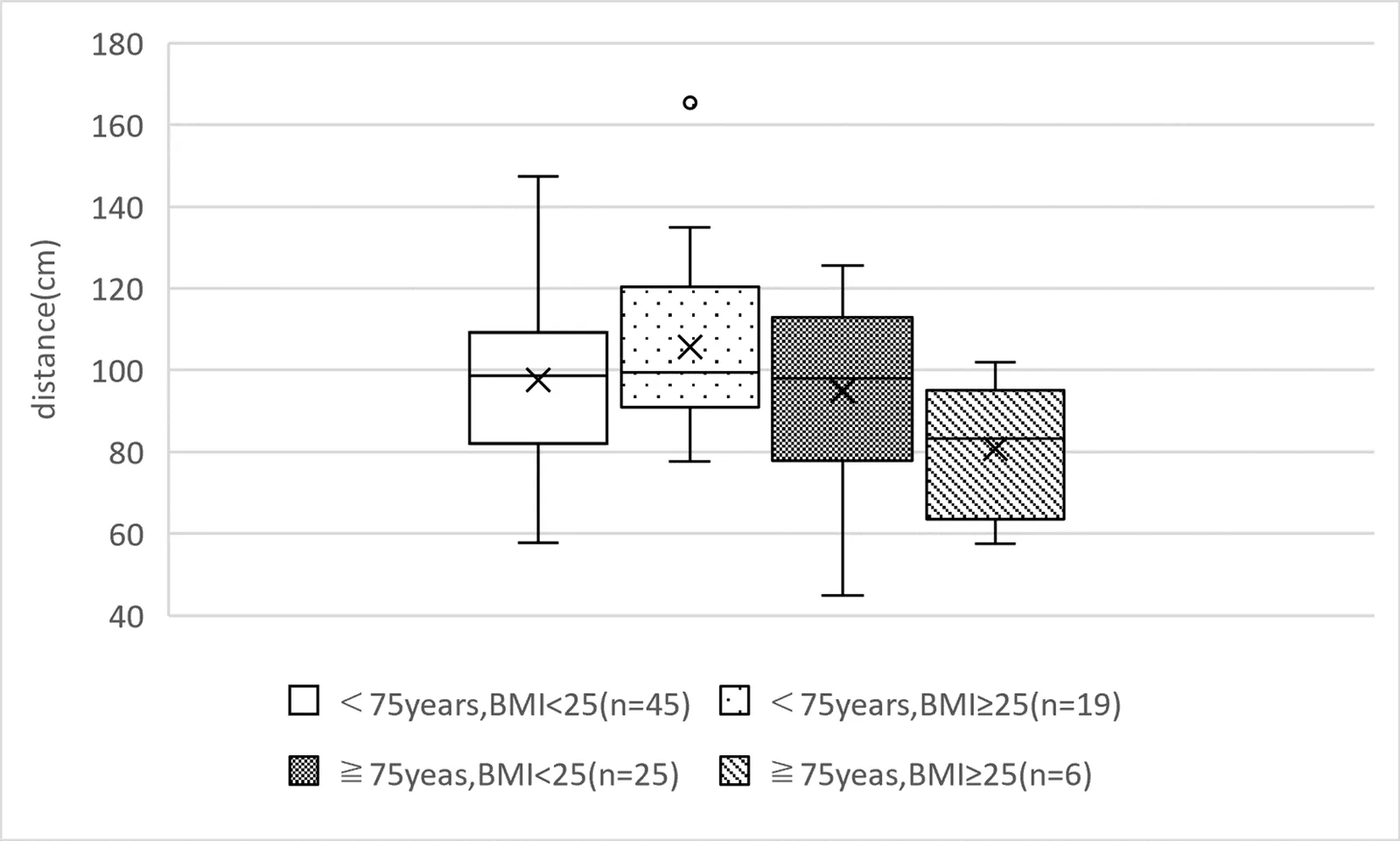
Comparison of optimal interpersonal distance from the pharmacist according to patient age and BMI The box plots show the distribution of preferred interpersonal distance in four groups according to patient age and BMI: age < 75 years/BMI < 25 kg/m², age < 75 years/BMI ≥ 25 kg/m², age ≥ 75 years/BMI < 25 kg/m², and age ≥ 75 years/BMI ≥ 25 kg/m². The horizontal line within each box represents the median, and the box represents the interquartile range. The cross indicates the mean, and circles indicate outliers. The four groups were compared using the Kruskal-Wallis test (χ²(3) = 5.785, p = 0.123). BMI: body mass index.

## Discussion

The present study investigated whether patient BMI was associated with preferred interpersonal distance from pharmacists during medication counseling. No statistically significant association between BMI and preferred interpersonal distance was observed in this sample. This finding was consistent when BMI was evaluated both categorically and as a continuous variable. These results suggest that body size alone may not be sufficient to predict the distance that a patient considers comfortable during communication with a pharmacist.

Personal space is influenced by multiple factors, rather than by a single physical characteristic. Previous studies have reported associations between interpersonal distance and sex, age, attractiveness, familiarity, and other individual characteristics [8-10]. Hall and Sommer emphasized that personal space is affected by social relationships, situations, and individual differences [6,7]. Therefore, even if the BMI reflects differences in body size, its influence may be less than that of psychological or social factors.

The perception of another person may also have an important influence on preferred interpersonal distance. Iachini et al. demonstrated that individuals maintain different distances, depending on their impressions of another person [12]. Similarly, Ruggiero et al. showed that facial expressions can modify interpersonal and peri-personal space [13]. Nose et al. reported the psychological and physiological responses when personal space was invaded and showed that these responses were influenced by the impression of the approaching person [11]. These findings indicate that interpersonal distance is strongly related to how another person is perceived.

This may be particularly relevant to pharmacist-patient communication. During medication counseling, patients may determine a comfortable distance based not only on their own physical characteristics, but also on the pharmacist's facial expression, posture, manner of speaking, familiarity, and perceived trustworthiness. Nonverbal behaviors are known to play an important role in communication [4,5]. Thus, these interpersonal factors may have a greater influence on the preferred distance than BMI.

Clinical studies also support the importance of individual variations in personal space. Research involving patients with schizophrenia has demonstrated differences in personal space in interview settings [14], and studies of nurse-patient interactions have suggested that a healthcare professional's position relative to the patient can influence comfort [15]. The spatial characteristics of the surrounding environment may also affect perceived interpersonal distance [16,17]. These observations suggest that an appropriate distance in healthcare settings should be considered dynamically rather than as fixed values.

Age may also be a relevant factor when considering interpersonal distance. A recent large-scale study demonstrated that interpersonal distance varies throughout a person’s lifespan [18]. Other studies have similarly reported age-related differences in interpersonal distance [10]. Therefore, patient age and other demographic or psychological characteristics may interact with situational factors and should be evaluated in future studies.

These findings have practical implications for pharmacy practice. Pharmacists are expected to provide patient-centered care and establish effective communication with the patients [1-3]. Rather than estimating a comfortable distance based on a patient's appearance or body size, pharmacists should observe the patient's reactions and adjust their positions accordingly. For example, signs such as leaning backwards, avoiding eye contact, changing posture, or moving away may indicate that the pharmacist is standing too close. Conversely, patients with difficulty in hearing or seeing medication materials may prefer shorter distances.

Therefore, it may be difficult to determine the appropriate interpersonal distance based solely on a patient's physical characteristics. Pharmacists should adjust their distance while observing each patient's response, and when appropriate, confirm the patient's preferences. Such an individualized approach may contribute to more comfortable pharmacist-patient communication.

This study has several limitations. First, this was a single-center study involving a relatively small sample of 95 outpatients receiving anticancer therapy, which may limit the generalizability of the findings. In addition, the BMI groups were unequal in size (BMI < 25 kg/m², n = 70; BMI ≥ 25 kg/m², n = 25), and the relatively small subgroup sizes may have limited the statistical power of the subgroup analyses. Second, BMI was categorized using a cutoff of 25 kg/m², and dichotomization may result in a loss of information. To address this limitation, we additionally analyzed BMI as a continuous variable using Spearman's rank correlation coefficient; however, no statistically significant correlation with optimal interpersonal distance was observed. Third, preferred interpersonal distance may be influenced by patient- and treatment-related factors that were not comprehensively evaluated in the present analysis, including personality, anxiety, visual impairment, familiarity with the pharmacist, duration of therapy, cancer type, cultural background, and previous healthcare experiences. Fourth, although the measurement procedure was standardized, measurements were performed by three pharmacists, which may have introduced some variability. Finally, interpersonal distance was measured under standardized conditions, including the supine position, a 45° angle between the patient and pharmacist, and aligned eye levels. Therefore, the distances measured under these controlled conditions may not fully reflect preferred interpersonal distance in other clinical settings or during routine pharmacy practice.

Future studies should include larger and more diverse populations and evaluate psychological, social, and environmental factors together with physical characteristics. It would also be useful to examine the interactions between interpersonal distance and nonverbal behaviors such as facial expressions, eye contact, posture, and body orientation. Such studies may help establish more individualized approaches to pharmacist-patient communication.

## Conclusions

This study investigated the association between patient BMI and preferred interpersonal distance from pharmacists among 95 outpatients receiving anticancer therapy. No statistically significant difference in optimal interpersonal distance was observed between patients with a BMI < 25 kg/m² and those with a BMI ≥ 25 kg/m². In addition, no statistically significant correlation was observed when BMI was analyzed as a continuous variable. No statistically significant differences were observed when BMI was analyzed in combination with age. Although the analysis combining BMI and sex showed a possible trend toward differences among the groups, this did not reach statistical significance.

These findings indicate that no statistically significant association between BMI and preferred interpersonal distance was identified in this sample. During medication counseling, pharmacists should consider individual patient characteristics and responses and adjust interpersonal distance accordingly. Further studies with larger and more diverse populations are needed to clarify the potential influence of BMI and its interactions with other patient characteristics.
